# Role of Diversity and Recombination in the Emergence of Chilli Leaf Curl Virus

**DOI:** 10.3390/pathogens11050529

**Published:** 2022-04-30

**Authors:** Megha Mishra, Rakesh Kumar Verma, Vineeta Pandey, Aarshi Srivastava, Pradeep Sharma, Rajarshi Gaur, Akhtar Ali

**Affiliations:** 1 Department of Biosciences, School of Liberal Arts and Sciences, Mody University of Science and Technology, Lakshmangarh, Sikar 332311, Rajasthan, India; meghamishra1228@gmail.com (M.M.); rkwat4@yahoo.com (R.K.V.); 2 Department of Biotechnology, D.D.U. Gorakhpur University, Gorakhpur 273006, Uttar Pradesh, India; vinita.pandey11@gmail.com (V.P.); sriaarshi090516@gmail.com (A.S.); 3 Department of Biotechnology, ICAR—Indian Institute of Wheat & Barley Research, Agarsain Road, Karnal 132001, Haryana, India; neprads@gmail.com; 4 Department of Biological Science, The University of Tulsa, 800 S Tucker Drive, Tulsa, OK 74104-3189, USA

**Keywords:** chilli leaf curl virus, recombination, mutation, genetic diversity, selection

## Abstract

Chilli leaf curl virus (ChiLCV), (Genus *Begomovirus*, family *Geminiviridae*) and associated satellites pose a serious threat to chilli production, worldwide. This study highlights the factors accountable for genetic diversity, recombination, and evolution of ChiLCV, and associated chilli leaf curl alphasatellite (ChiLCA) and chilli leaf curl betasatellite (ChiLCB). Phylogenetic analysis of complete genome (DNA-A) sequences of 132 ChiLCV isolates from five countries downloaded from NCBI database clustered into three major clades and showed high population diversity. The dN/dS ratio and Tajima D value of all viral DNA-A and associated betasatellite showed selective control on evolutionary relationships. Negative values of neutrality tests indicated purified selection and an excess of low-frequency polymorphism. Nucleotide diversity (π) for C4 and Rep genes was higher than other genes of ChiLCV with an average value of π = 18.37 × 10^−2^ and π = 17.52 × 10^−2^ respectively. A high number of mutations were detected in TrAP and Rep genes, while ChiLCB has a greater number of mutations than ChiLCA. In addition, significant recombination breakpoints were detected in all regions of ChiLCV genome, ChiLCB and, ChiLCA. Our findings indicate that ChiLCV has the potential for rapid evolution and adaptation to a range of geographic conditions and could be adopted to infect a wide range of crops, including diverse chilli cultivars.

## 1. Introduction

Viruses and diseases that have emerged in the past few years have impeded the production of important crops worldwide. Genetic diversity allows the adaptation of virus populations to a varying environment. Many virus species are the result of closely related genomic variants due to a high rate of mutations, rapid recombination, and a large population size [1]. Research on the evolution of plant viruses is mainly concentrated on RNA viruses, but very few studies exist on DNA viruses, although (ss) DNA viruses are the biggest emerging menace to agriculture globally. Several studies have revealed that ssDNA viruses may evolve as quickly as RNA viruses [2]. A number of studies anticipated that ssDNA viruses possibly replicate through low-fidelity DNA polymerases, and those spontaneous biochemical reactions which preferably act on ssDNA (methylation, deamination and oxidation of bases) might be contributing to the genetic variations of ssDNA viruses [3,4]. Though mutation dynamics acts as the most important factor for virus diversification [5], it does not describe all the standing genetic variation. Another crucial factor that significantly contributes to diversity of plant viruses is recombination. Recombination contributes to the evolution of a number of virus species and has been extensively identified in the genomes of many geminiviruses [1,6,7,8].

The genus *Begomovirus* comprises more than 400 speciesand is the largest genus in the family *Geminiviridae* which are transmitted by whiteflies (*Bemisia tabaci)* [9,10]. Based on pair-wise sequence identity, host range, genome-organization and insect-vector relationship, the family *Geminiviridae* has been divided into nine genera: *Becurtovirus*, *Begomovirus*, *Capulavirus*, *Curtovirus*, *Eragrovirus*, *Grablovirus*, *Mastrevirus*, *Topocuvirus* and *Turncurtovirus* [9,11,12]. Recently, five more genera: *Citlodavirus*, *Maldovirus*, *Mulcrilevirus*, *Opunvirus* and *Topilevirus* have been added thus increasing the total number to 14 genera in the *Geminiviridae* family [13].Begomoviruses are ssDNA plant viruses having geminate quasi-icosahedral virions [9]. The genome of begomovirus can be monopartite (DNA-A) or bipartite (DNA-A and DNA-B) of ~2.5–2.7 kb per genome component in size. DNA-A encodes six virus proteins. Four proteins are encoded by the complementary sense strand: replication associated protein (Rep/AC1), transcription activator protein (TrAP/AC2), replication enhancer protein (REn/AC3) and AC4 while virions sense strand encodes for coat protein (CP/AV1) and pre-coat protein (pre-CP/AV2), in some begomoviruses species AC5/C5 protein were also observed [6,14]. DNA-B is involved in the systemic (cell to cell) and local (nucleus to cytoplasm) movements of begomovirus and involved proteins aremovement protein (MP) and nuclear shuttle protein (NSP) respectively [10]. The alphasatellites and betasatellites are predominantly associated with monopartite begomoviruses [15,16] and a few cases have been reported for deltasatellites [17]. The alphasatellite belongs to family *Alphasatellitidae*, encoding a single protein alpha-rep and includes a hairpin structure at the origin of replication [15]. The betasatellite is about half the size of begomovirus DNA-A, encodes the βC1protein in the complementary sense strand, and plays important roles in transcriptional and post transcriptional gene silencing and symptom induction [18,19].

Chilli (*Capsicum annuum*) is a vital spice in Indian cuisine or food which is used both as a fresh vegetable and in powder form. The involvement of numerous begomoviruses (including ChiLCV) and associated DNA satellites in Chilli infection and the development of Chilli leaf curl disease were reported several years ago [14]. Chilli leaf curl virus (ChiLCV) is among the most predominant monopartite begomoviruses and seriously impacts solanaceous and non-solanaceous hosts in combination with various betasatellites [20]. Due to ChiLCV infection, 14–100% yield losses of chilli were recorded in Rajasthan (India) [21] and causes severe economic losses in both the tropical and sub-tropical regions of India [14,21]. The typical symptoms caused by the virus infection in chilli are leaf curling, crumpling, thickening and swelling of the veins, reduced leaf size, shortening of internodes and petioles, clustering of leaves and stunning of whole plant [12]. ChiLCV has a wide host range and could pose the possibility of an outbreak in the Indian sub-continent. However, numerous site-specific nucleases have been developed for direct interference with the begomovirus genome [22,23].

In this study, we analysed the genetic diversity of a large number of ChiLCV isolates from five countries and its satellite populations to get a better insight into the epidemics, and evolution of ChiLCV. We further analysed the phylogenetic relationship, and recombination breakpoints in the complete genome sequences of ChiLCV isolates, as well as their associated satellites viruses, and significant findings are outlined in this article.

## 2. Results

### 2.1. Phylogenetics and Estimation of Nucleotide Substitution Rates

**Figure 1:**
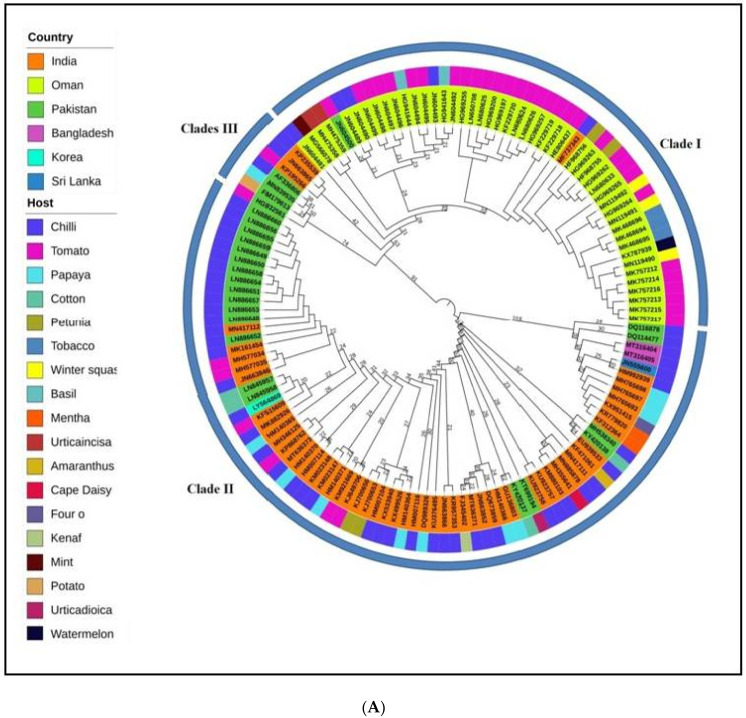

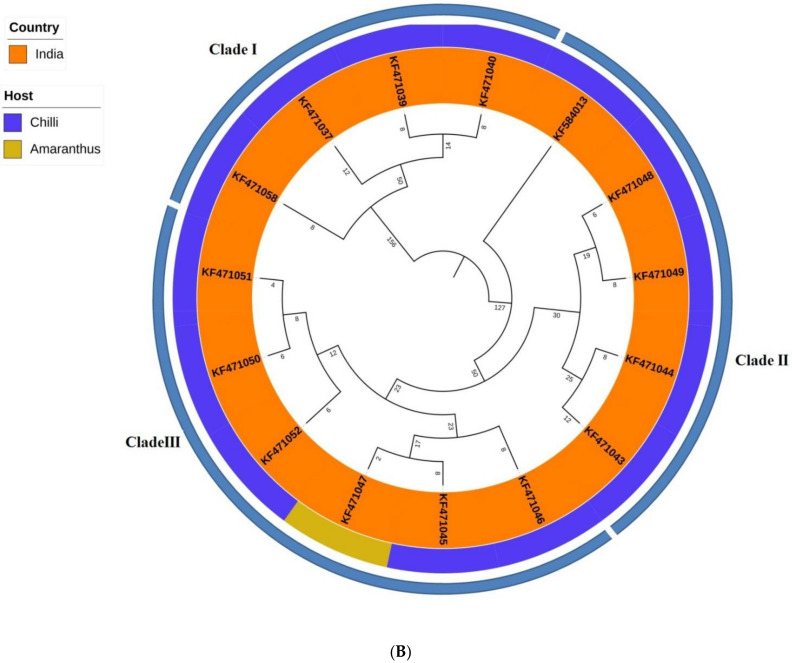

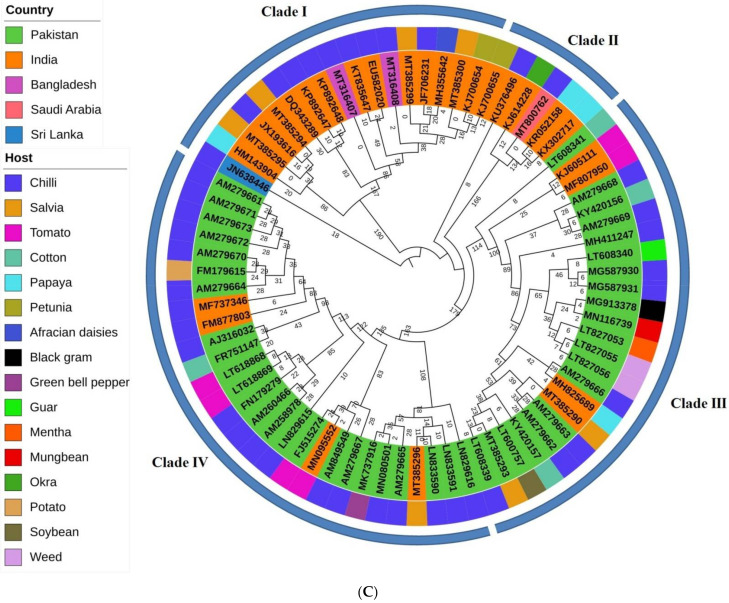
Bayesian method and Tree annotator tool available in BEAST v. 1.10 v. 1.10 [24] were used for maximum clade credibility (MCC) phylogenetic analyses based on full-length nucleotide sequences of ChiLCV and associated satellites with rooted tree mid-point and the tree was built in Interactive Tree Of Life (iTOL) an online tool (numbers indicates the height median for each isolates and associated satellites). The phylogenetic trees indicate to check the evolutionary relatedness among the virus populations of (**A**) Chilli leaf curl virus (ChilCV), (**B**) Chilli leaf curl *alphasatellite* (ChiLCA) and (**C**) Chilli leaf curl *betasatellite* (ChiLCB). The outermost ring shows the clades formation among the viruses whereas the middle ring indicates the host and the innermost ring shows country of origin.

Using the complete genome of DNA-A of ChiLCV, maximum clade credibility phylogenetic analysis was performed to check the evolutionary relatedness among the virus populations. A total of 132 ChiLCV isolates reported from India, Pakistan, Oman, Sri Lanka, Bangladesh and Republic of Korea were grouped into three distinct clades (I, II and III). Majority of isolates in clade I were from Oman (28) whereas clade II indicates isolates from India (50), Pakistan (21), one from both Bangladesh and South Korea along with a single isolate from Sri Lanka also shared the same clade (Figure 1A). Moreover, in clade III majority of isolates were from Pakistan (04 isolates) and India (03) (Figure 1A). Additionally, this phylogenetic analysis was reinforced by nucleotide sequence identity test through Sequence Demarcation Tool Version 1.2 (SDTv1.2) (Supplementary Figure S1) that aids to interpret the phylogenetic tree analytically and efficiently.

The fifteen isolates of alphasatellites were grouped into three different clades (Figure 1B). All clades consist of isolates belong to Indian origin associated with chilli crop except KF471047, which was associated with *Amaranthus* crop from India belonging to Clade III. The ChiLCA sequence KF584013 seems somewhat different among Clade II sequences, the key reason behind this out-grouping was its least nucleotide sequence identity (<78%) with all other ChiLCA sequences (Supplementary Figure S2) which was might be a consequence of frequent recombination and mutation events. On the other hand, the isolates of betasatellites were divided into four clades (Figure 1C). Clade I had all isolates obtained from India (fourteen isolates), Bangladesh (two isolates) and Sri Lanka (one isolates), whereas majority of isolates groped in Clade II (five isolates) were from India except MT800762 which was from Saudi Arabia. While considering Clade III (twenty-four isolates) and Clade IV (twenty-eight isolates), among them we found that the majority of isolates were from Pakistan. In ChiLCB isolate, JN638446 (Sri Lanka) had the least sequence nucleotide identity (<70%) for all other ChiLCB sequences, perhaps because (Supplementary Figure S3) clades owe their phylogenetic relationships to other sequences. This phylogenetic finding facilitates the interpretation of evolutionary patterns existing among ChiLCV and its associated satellite populations across major different regions of the world, and although the isolates of different countries share the same clades pointing us in a direction that indicates the cross-border movement of these virus isolates.

The overall rate of nucleotide substitution was 3.34 × 10^−3^ substitutions/site/year (with 95% HPD interval 1.72 × 10^−3^ to 6.62 × 10^−3^) for DNA-A of ChiLCV, which is very much similar to the nucleotide substitution rate of plant RNA viruses demonstrating a rapid rate of evolution. In addition, the average rate of nucleotide substitution for alphasatellites and betasatellites was 6.19 × 10^−3^ substitutions/site/year and 9.17 × 10^−4^ substitutions/site/year respectively (Table 1).

The overall rate of evolution of all individual genes of DNA-A component exhibited a high rate of nucleotide substitution. The mutation rate (an important parameter for the calculation of the rate of evolution) of all the three codon positions among all the ORFs was observed in the CP gene at codon position 3 and for Pre-CP and C4 genes at codon position 2. Similarly, both alphasatellite and betasatellite have a high mutation rate at codon position 2. The other datasets (pre-CP and CP) had a higher mutation rate for the 2nd and 3rd codon positions (Table 1).

### 2.2. Recombination Analysis

**Figure 2:**
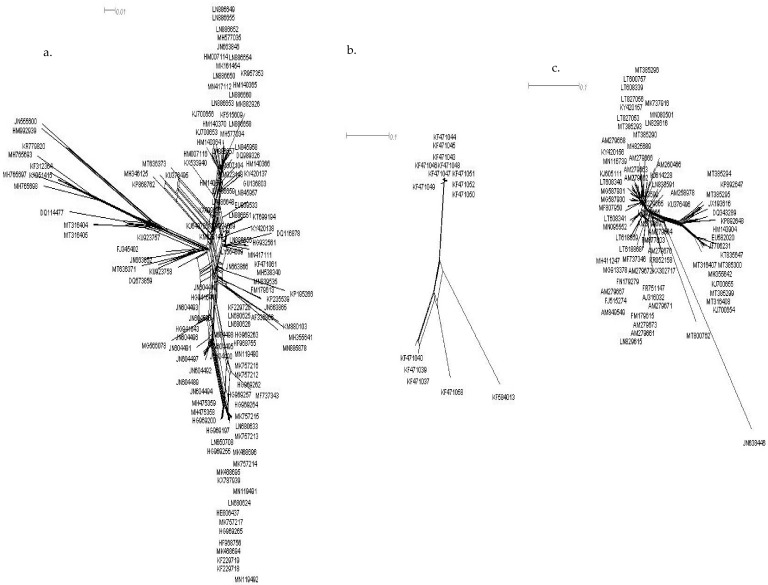
Reticulate network showing the presence of recombination events of ChiLCV (**a**) DNA-A (**b**) alphasatellite (**c**) betasatellite.

Phylogenetic networks showing reticulation (Figure 2) demonstrate clear evidence for recombination events. Putative recombination breakpoints are analysed by the RDP v. 4.2 package [25]. Analysis identified many unique recombination events in all the datasets. To avoid unreliable signals, only recombination events supported by at least three or more different methods with significant support were selected. Fifty-one recombination breakpoints were observed in ChiLCV DNA-A, mostly located in the C1 gene of the C-sense strand and the V1 gene of the V-sense strand (Table 2a). These results were further confirmed by recombination breakpoint analysis for each ORF (C1, C2, C3, C4, V1 and V2) of DNA-A. The Rep gene (C1) was identified as the main contributing factor that was involved in intra-species recombination with fifteen breakpoints. Table 2b. CP (V1) regions showed five recombination breaks, and TrAP (C2) and Ren (C3) regions also showed three recombination breakpoints each. The putative recombination analysis for alphasatellite and betasatellite of ChiLCV showed that betasatellite has higher recombination events, i.e., seventeen recombination events more than alphasatellite (Table 2b).

**Figure 3:**
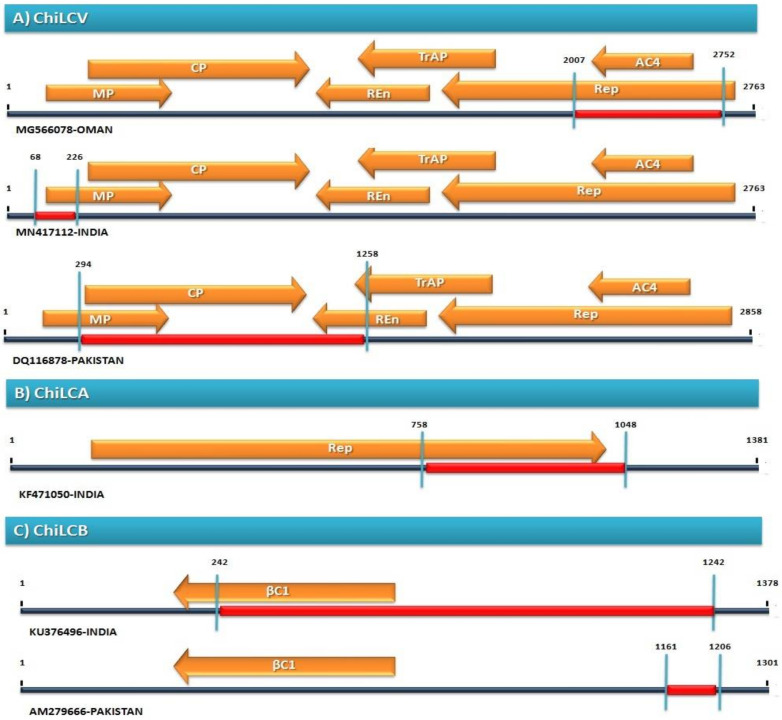
Frequently detected recombination events among populations of different countries (**A**) ChiLCV, (**B**) ChiLCA and (**C**) ChiLCB. Red line highlights the genome area exhibiting recombination.

**Figure 4:**
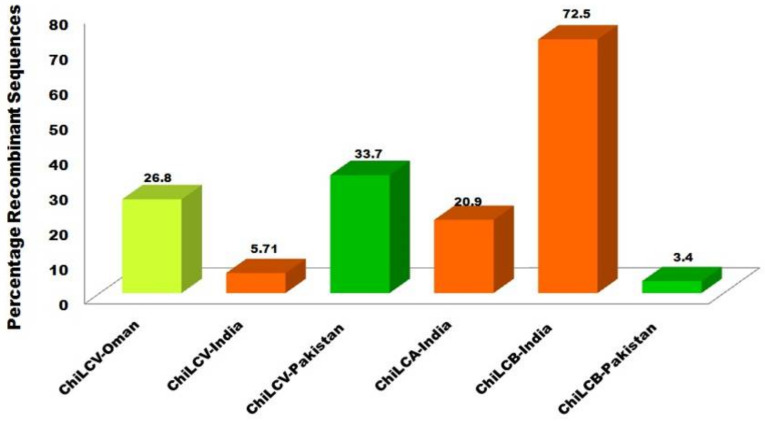
Evaluation of percentage sequences among ChiLCV, ChiLCA, and ChiLCB developing through recombinational variation with different locations of geographical origin.

A dataset of ChiLCV isolates from Oman, India, and Pakistan was observed for frequently recombination events and we found that an isolate ChiLCV-MV3(MG566078,Oman), ChiLCV-CHL46 (MN417112,India) and PepLCPV-Khanewal 1 (DQ116878,Pakistan) contribute to frequent recombination by 49, 17, and 1 event out of 60 recombination events (*p*-value = 5.50 × 10^−29^, 6.15 × 10^−05^, 4.58 × 10^−66^) at positions 2007–2752, 68–226, and 294–1258 respectively (Figure 3A, Table 2a). Meanwhile, among above analyzed sequences, we identified that a significant variation exists amid viral populations. For instance, for isolate ChiLCV-MV3 (MG566078, Oman), recombination enclosed almost 26.8% of the ChiLCV genome (Figure 3A, Figure 4). The *Rep* genes had partial covered while in MN417112-India only 5.7% of ChiLCV (Figure 3) genome coverage was detected by recombination slightly enclosed *MP* genes. However, in PepLCPV-Khanewal 1 (DQ116878, Pakistan), recombination covers almost 33.7% of the ChiLCV genome (Figure 4) with complete coverage of *CP* genes and *MP* and REn genes was partially covered (Figure 3A). Moreover, while studying ChiLCA (20.9% coverage) the sequences was only reported from India, maximum recombination breakpoint was found in KF471050 at 758–1048 position (Figure 3B, Table 2b) that covers the partial region of *Rep* gene. Simultaneously, in ChiLCB, isolates from India and Pakistan were detected for frequently recombination events for ChiLCB-CNB (KU376496, India) and ChiLCB-chM34 (AM279666, Pakistan) at 242–1242 and 1161–1206 position respectively (*p*-value = 1.64 × 10^−09^ and 7.57 × 10^−11^) (Figure 3C and Figure 4, Table 2a) while in KU376496-India it exhibited recombinant sequences covering 72.5% of whole genome that encloses maximum coding region i.e., βC1 which is comparatively not only more than ChiLCB-chM34 (AM279666, Pakistan) but also highest among all other ChiLCB isolates, thus this was one of the main reasons for the out-grouping of the KU376496-India isolate in Clade II (Figure 3C).

### 2.3. Population Demography Analysis

The total number of mutations was η = 2104 in DNA-A, with the highest number of mutations in TrAP (426 nt) and Rep (399 nt) genes indicating that the diversification of the ChiLCV population is mainly driven by mutation in these two genes. ChiLCB has 1070 mutations, while ChiLCA has 727 mutations. ChiLCV DNA-A has a high degree of genetic variability (π > 0.08) i.e., π = 0.107, along with both the satellite molecules. Among all the ORFs of DNA-A, C4 (π = 0.183) and Rep (π = 0.175) gene had the highest nucleotide diversity (Table 3). Haplotype distribution analysis revealed different values among the 132 ChiLCV isolates, based on the six coding regions evaluated. Among the total sequences of ChiLCV (*n* = 132), the number of haplotypes ranged from 55 in pre-CP and C4 regions to 81 in CP region, with maximum haplotypes 113 in whole genome. Each isolate represented a maximum number of haplotypes at the CP region, showing high genetic variation within the coding gene.

Neutrality tests (Tajima’s D, Fu and Li’s *D* and Fu and Li’s *F*) were performed to examine the evidence of demographic forces or selection acting on ChiLCV population and satellite molecules. Negative Tajima’s *D* values were obtained in all ORFs encoded by DNA-A in analyzed dataset (Table 4) suggesting a purifying selection and population expansion [26]. Similarly, for all the virus datasets statistical parameters like Fu and Li’s *D* and Fu and Li’s *F* tests, we have obtained negative and, in some case, positive values (Table 4) reiterating the operation of purifying selection and population expansion that possibly have played a role in the observed diversity. The combination of negative values of Tajima’s *D*, Fu and Li’s *D* and Fu and Li’s *F* values signify that ChiLCV population is under purifying selection.

### 2.4. Amino Acid Sites under Selections

The calculated dN/dS ratio was >1 for DNA-A, CP and pre-CP genes and 1.081 and 1.309 for ChiLCA and ChiLCB respectively (Table 4), demonstrating the prevalence of diversifying selection acting on virus genome and selected individual genes. These results indicate other genomic component (REP, TrAP, REn and C4) sites are under negative selection. However, the results showed a wide range amongst six genes/datasets (0.448 to 1.131) of ChiLCV, representing diverse selective constraints in different datasets. The CP and pre-CP gene was under stronger negative selection as compared to all other genes in the DNA-A of ChiLCV.

Fewer sites of the virus from all over the genome showed evidence of positive selection for all the datasets. For ChiLCV DNA-A, 101 positive selective sites were calculated. Among all the genes, Rep (17 sites) and TrAP (13 sites) have the maximum sites under positive selection. Positive selection sites were also detected in ChiLCB (34 sites) and ChiLCA (3 sites) (Table 4).

## 3. Discussion

ChiLCV is one of the most damaging begomoviruses and causes significant yield loss es in chilli production, worldwide. Due to the mixed cropping system and the polyphagous nature of the vector “whitefly”, it leads to an overlapping host range of begomoviruses. ChiLCV has a wide host range and infects chilli, papaya, tomato, eggplant, hibiscus etc. [20]. The genetic structure of ChiLCV DNA-A with all six ORFs and associated satellite molecules was evaluated. This study elucidates the evolution and variability of the ChiLCV (whole genome and individual ORFs) and associated satellite molecules by using dated published genomic sequences. The two major factors contributing to the high genetic variability of begomoviruses are frequent recombination, which might considerably accelerate their evolution by increasing the permutations of pre-existing nucleotide polymorphism generated by mutations [8,27] and the high nucleotide substitution rate as rapidly as most RNA viruses [3,4,28]. Therefore, mutation and recombination are considered crucial factors involved in the genetic variability of ChiLCV populations [1,5,29]. In this study, we have analysed various parameters for ChiLCV isolates and shown which genes are more affected by the above factors. Satellite molecules (alphasatellite and betasatellite) are essential pathogenicity determinants for monopartite begomoviruses and have also been investigated [30,31].

The phylogenetic studies estimated from the whole-genome sequences of ChiLCV and associated satellite molecules were qualitatively congruent. The phylogenetic tree of DNA-A component represents a strong association of ChiLCV Indian isolates with isolates of Pakistan, Oman, and Sri Lanka (Figure 1A). The phylogenetic relationship indicates that Indian isolates of ChiLCV played an important role in the evolution of the virus and are involved in intra-species recombination of ChiLCV isolates. The movement of whiteflies from India to the adjacent countries and similar cropping patterns may be the possible explanation for the intra-species recombination and phylogenetic relationship.

Analysis of homology and phylogeny further highlighted the evolutionary relatedness among ChiLCV populations arising from different countries. For instance, Indian ChiLCV isolates clustered with Pakistani isolates in clades II and III, whereas clade I contain mainly isolates from Oman. Furthermore, the emergence of new isolates of ChiLCV in the past era is now quite alarming for agriculture production as this virus is expanding the host range. Therefore, while the expanding host range of viruses is imperative to assess their evolutionary mechanisms; the diversity and genetic structure of viral populations in a single host are equally important to explain the evolutionary patterns [32]. The dominance of alphasatellites in India and betasatellites in Pakistan and India as compared to other regions might be attributed to the presence of suitable hosts and efficient transmission vectors [33]. To date, there is no evidence of chilli leaf alphasatellites from Pakistan, Oman, Sri Lanka, Bangladesh, or the Republic of Korea satellite. The majority of ChiLCA, found to be associated with chilli (excluding one host for Amaranthus, KF471047), explains the absence of suitable hosts in these regions. However, only one isolate of ChiLCB (JN638446; 2011) has been reported from Sri Lanka (Supplementary Table S1), the possible emergence of ChiLCV-associated satellites in the future cannot be neglected. Over the past few years, multiple infections of satellites were found to be associated with the ChiLCD complex, and additionally, alphasatellites were found in co-existence with betasatellites [34]. Because of the prevalent occurrence of mixed infection among begomoviruses, finding several and co-existing satellites may not be unusual [35]. Perhaps during whitefly-mediated transmission, satellite molecules might become associated with other viruses, forming new complexes and introducing them to disease-free regions. The two ChiLCA (KF584013 and KF471058) from India and the ChiLCB sequences JN638446 (SriLanka) showed a distinct outgroup with other clades, highlighting the importance of component recombination and reassortments.

Population genetics, along with recombination, are important factors influencing DNA virus evolution. Mutation plays a crucial role in genetic variation, on which recombination, natural selection, genetic drift, and gene flow act to shape the genetic structure of population [1], as shown in this work and previous studies with other plant viruses [12]. The isolates of ChiLCV exhibited a non-recombination structure in a more diversified form due to occurrence of maximum mutation then those to recombinant region. Hence, we found that the most viable recombinant gene, i.e., C1 has a low number of mutation sites shows high recombination breakpoints compared to C2 gene having a high number of mutation sites, shows low recombinant breakpoints. Therefore, the detected recombination patterns consequently seemed to have diverged from each other by point mutation, which highlights the genetic distribution likely involves the contribution of mutation in facilitating virus evolution.

Recombination among the sequence of all datasets might provide a high rate of evolution, rapid multiplication, and expansion of the host range. Various studies have revealed the high frequencies of recombination in begomovirus populations [36] and for ssDNA viruses which use a rolling circle replication mechanism, non-random sites of recombination events are a conserved trait [6,7,37]. In this study we observed at least one recombination breakpoint in analyzed datasets of DNA-A component and associated satellite molecules which strengthen the previous studies. The high recombination frequency in begomoviruses could leads to the emergence of new begomovirus species and helps to acquire satellite molecules [38]. In our dataset of ChiLCV we observed forty-seven recombination breakpoints with more than three algorithms implemented in RDP v. 4.2, suggests high genetic variation in ChiLCV genome (Table 1). Recombination in DNA-A and DNA-B components in bipartite begomoviruses and recombination with associated satellite molecules in monopartite begomoviruses were also reported along with intra-species recombination [39]. The Rep and CP gene in begomoviruses exhibits higher number of recombination as compare to other genes. This uneven presence of recombination events in begomoviruses genes supports that it is a major factor for genetic variation in begomoviruses. In our dataset of ChiLCV we observed the Rep and CP gene in begomoviruses exhibit higher number of recombination event as compared to other genes of DNA-A component. The associated betasatellite also showed higher number of recombination breakpoints than the alphasatellite (Table 2). The Recombination analysis results obtained in this study also supports that the recombination is a driving force of the genetic variability in ChiLCV genome.

Numerous studies reported that the geminiviruses have a high nucleotide substitution rate, which is almost analogous to those of RNA viruses [14]. Here, the observed nucleotide substitution rate is higher than those considered for double-stranded DNA viruses [3]. The wide range of dN/dS values in a population implies that the populations may be under the influence of purifying selection or have experienced recent expansion [40]. In this study we observed the higher dN/dS ratio in CP and Pre-CP gene and lower dN/dS ratio in Rep and C4 genes of DNA-A component of ChiLCV (Table 4) revealing the occurrence of diversifying selection acting on virus genome. The wide range of dN/dS ratio in ChiLCV analyzed dataset (Table 4) demonstrates the presence of purifying selection and exhibits the strong negative selection in CP and Pre-CP gene of ChiLCV. As previous studies have already shown that in begomoviruses the most of sites are under purifying selection pressure, and few sporadic sites were identified as experiencing positive selection [41]. In our dataset we observed fewer sites in the ChiLCV are under positive selection. Positive selection sites were also detected in ChiLCB (34 sites) and ChiLCA (3 sites) (Table 4).Our results support the fact that the positive selection is also acting as a major pressure responsible for the increased levels of genetic diversity in ChiLCV isolates.

In summary, this study further confirmed that ChiLCV populations are mostly influenced by mutation and recombination, which play a crucial role in the genetic diversification of the ChiLCV population. However, it needs to be determined how mutations are influenced by diverse hosts infected by ChiLCV. As we know that the chilli leaf curl disease caused by various begomoviruses involves enormous losses in chilli cultivation, worldwide and a matter of great concern for farmers as well as agricultural scientist. To combat this there is a great need of more studies related to evolving nature of ChiLCV. This study includes the analysis of major evolutionary driving forces such as mutation, recombination and natural selection in ChiLCV population, which helps us to understand the genetic variability in ChiLCV and to develop new strategies to control viral diseases in chilli and other susceptible crops.

## 4. Material and Methods

### 4.1. Sequence Datasets and Multiple Sequence Alignments

Complete genome sequences (DNA-A) of 132 isolates of ChiLCV in which 54 isolates were reported from India, 49 from Oman, 25 from Pakistan, 2 from Bangladesh and one each from Korea and Sri Lanka, 75 complete sequences of ChiLCB and 15 of ChiLCA were retrieved from the NCBI GenBank database using the Taxonomy Browser (www.ncbi.nlm.nih.gov, accessed on 6 October 2021). Along with whole-genome sequences of ChiLCV and satellite molecules, multiple sequence alignments for all the six genes of DNA-A were also analysed using the Muscle algorithm implemented in MEGA X [42].

### 4.2. Phylogenetic and Coalescent Analysis

The Maximum clade credibility phylogenetic tree was constructed by using, the Bayesian method and Tree annotator tool available in BEAST v. 1.10 [43]. To select the best-suited nucleotide substitution model for each dataset MEGA X [42] had been employed. The resulting trees were visualized and edited in iTOL v6.5 (Interactive Tree Of Life) (https://itol.embl.de/#, accessed on 13 April 2022) [44]. To estimate the nucleotide substitution rates per site and mutations at various codon positions, the Bayesian Markov Chain Monte Carlo (MCMC) method obtainable in BEAST v. 1.10 [43] was used. Each data set was analysed by both relaxed and strict molecular clocks (uncorrelated exponential and uncorrelated lognormal). MCMC chains were run for sufficient length (10^7^) and statistical uncertainty in the estimates was provided by the 95% highest probability density (HPD) value. Best-fit clock and coalescent constant demographic models were identified and achievement of suitable effective sample sizes for these parameters was estimated by using Tracer v1.5 [44].

### 4.3. Recombination Analysis

Phylogenetic network analysis was performed for evidence of recombination with the neighbor net method implemented in Splits Tree 4 [45]. To identify the parental isolates to substantiate the recombination events, breakpoints and origin of the virus spread were predicted by using RDP, GENECONV, MAXCHI, BOOTSCAN, CHIMAERA, SISCAN and 3SEQ methods implemented in RDP v. 4.2 [25] with default detection thresholds and 0.05 highest acceptable Bonferroni corrected *p*-value.

### 4.4. Population Demography Analysis

To investigate the nucleotide polymorphism various parameters were calculated by using DnaSP v. 6.0 [46]. The estimation of genetic diversity was determined by the number of polymorphic sites (S), total number of mutations (η), nucleotide diversity (π), number of haplotypes (h), haplotype diversity (Hd), Watterson’s estimate of the population mutation rate based on the total number of segregation sites (θ − w) and the total number of mutations (θ − η). Neutrality tests were also performed using Tajima’s *D* (nucleotide diversity with the proportion of polymorphic sites), Fu and Li’s D* (difference between the number of singletons and the total number of mutations) and Fu and Li’s F* (difference between the number of singletons and the average number of nucleotide differences between paired sequences) tests available in DnaSP v. 6.0.

### 4.5. Detection of Positive and Negative Selection at Amino Acid Sites

The ratio of non-synonymous to synonymous (dN/dS) substitutions was calculated by using standard parameters in MEGA X for every dataset. Ratio dN/dS > 1, dN/dS < 1, and dN/dS = 1 indicates positive (diversifying), negative (purifying), and neutral selection pressure, respectively. The detection of sites evolved under positive and negative selection was performed by three methods: single-likelihood ancestor counting (SLAC), fixed-effects likelihood (FEL) and fast unbiased Bayesian approximation (FUBAR) implemented in the DataMonkey (www.datamonkey.org, accessed on 19 March 2022) [47].

## Figures and Tables

**Table 1:** Mean substitution rate estimation of ChiLCV DNA-A, ChiLCA and ChiLCB.

Viral Component		DNA-A	CP	Pre-CP	Rep	TrAP	REn	C4	Alphasatellite	Betasatellite
**Mean substitution rate** **(at 95% HPD interval)**	**Relaxed clock**	3.34 × 10^−3^ (1.72 × 10^−3^, 6.62 × 10^−3^)	1.00 × 10^−2^ (1.64 × 10^−3^, 0.027)	0.012 (1.56 × 10^−3^, 0.030)	2.72 × 10^−3^ (1.11 × 10^−3^, 5.76 × 10^−3^)	5.75 × 10^−3^ (2.25 × 10^−3^, 0.011)	3.58 × 10^−3^ (1.06 × 10^−3^, 6.51 × 10^−3^)	4.08 × 10^−3^ (5.52 × 10^−5^, 0.03)	6.19 × 10^−3^ (4.11 × 10^−6^, 0.016)	9.17 × 10^−4^ (4.92 × 10^−4^, 1.35 × 10^−3^)
	**Strict clock**	6.17 × 10^−4^ (4.67 × 10^−4^, 8.29 × 10^−4^)	6.57 × 10^−9^ (2.34 × 10^−17^, 4.31 × 10^−8^)	1.11 × 10^−4^ (2.15 × 10^−9^, 2.55 × 10^−4^)	3.95 × 10^−4^ (2.73 × 10^−4^, 5.49 × 10^−4^)	3.64 × 10^−4^ (1.79 × 10^−4^, 5.28 × 10^−4^)	3.41 × 10^−4^ (1.43 × 10^−4^, 5.50 × 10^−4^)	1.12 × 10^−4^ (3.037 × 10^−11^, 3.69 × 10^−4^)	4.23 × 10^−5^ (1.10 × 10^−18^, 2.56 × 10^−4^)	1.06 × 10^−3^ (8.47 × 10^−4^, 1.279 × 10^−3^)
**Mutation at 3 different codon positions** **(CP1, CP2, CP3 respectively)**	**Relaxed clock**	0.92, 1.13, 0.86	0.58, 0.32, 2.10	0.41, 1.44, 1.16	1.32, 0.85, 0.83	0.97, 0.87, 1.17	1.02, 0.69, 1.11	0.84, 1.17, 0.99	1.05, 1.09, 0.85	0.99, 1.09, 0.91
	**Strict clock**	0.97, 1.20, 0.88	0.61, 0.36, 2.05	0.47, 1.39, 1.14	1.31, 0.87, 0.824	0.93, 0.84, 1.23	1.16, 1.03, 0.81	0.85, 1.67, 0.98	1.06, 1.11, 0.83	0.992, 1.10, 0.91

**Table 2:** a: Putative recombination events detected among ChiLCV isolates calculated by different algorithms. b: Putative recombination breakpoints in all the six genes of DNA-A ChiLCV and satellite molecules.

**a**							
**Events**	**Breakpoints**		**Recombinant**	**Parents**		**Methods ^$^**	***p*-Value ^#^**
	**Begin**	**End**		**Major**	**Minor**		
**ChiLCV DNA-A**							
1	294	1258	DQ116878_PepLC	MH538340__ChiL	DQ114477_ChiLC	**R**, G, B, M, C, S, 3S	4.58 × 10^−66^
2	522	1032	JN604496_ChiLC	KF229719_ChiLC	MK882926_ChiLC	**R**, G, B, M, C, S, 3S	3.68 × 10^−47^
3	2704	537	KM880103_ChiLC	MF737343_ChiLC	Unknown (MT636373_ChiLC)	**R**, G, M, C, S, 3S	4.54 × 10^−40^
4	2748	632	HM140366_ChiLC	HM007104_ChiLC	Unknown (KP195266__ChiL)	R, G, B, M, C, S, **3S**	3.22 × 10^−36^
5	2741	958	KP195266__ChiL	HG969255__ChiL	KU376495_ChiLC	R, **G**, B, M, C, S, 3S	4.69 × 10^−33^
6	2348	2752	KP868762_ChiLC	Unknown (MN417111_ChiLC)	HM007116_ChiLC	**R**, G, B, M, C, S, 3S	3.82 × 10^−31^
7	2206	2720	EU939533_ChiLC	HM140366_ChiLC	Unknown (DQ673859_ChiLC)	R, G, B, M, C, S, **3S**	1.16 × 10^−44^
8	2111	2590	DQ114477_ChiLC	KP235539_ChiLC	HM992939_ChiLC	**R**, G, M, C, S, 3S	1.21 × 10^−27^
9	521	1001	KP235539_ChiLC	KF229719_ChiLC	MK882926_ChiLC	R, **G**, M, C, S, 3S	1.07 × 10^−27^
10	56	614	KP868762_ChiLC	MT636373_ChiLC	MK882926_ChiLC	R, **G**, B, M, C, S, 3S	1.81 × 10^−26^
11	538	962	KF229719_ChiLC	MH355641_ChiLC	Unknown (HM992939_ChiLC)	R, **G**, M, C, S, 3S	3.21 × 10^−26^
12	2145	2652	HM992939_ChiLC	Unknown (KU923758__ChiL)	MH765698__ChiL	R, **G**, M, C, S, 3S	2.86 × 10^−23^
13	1378	1542	KJ700656_ChiLC	MH577034_ChiLC	Unknown (FJ345402_ChiLC)	R, **G**, B, M, C, S, 3S	4.80 × 10^−22^
14	479	1029	KU923757__ChiL	KU923758__ChiL	Unknown (MK882926_ChiLC)	R, G, M, C, S, **3S**	2.50 × 10^−24^
15	1030	1178	MH765697_ChiLC	Unknown (KM880103_ChiLC)	JN604491_ChiLC	**R**, G, M, C, S, 3S	1.14 × 10^−20^
16	2007	2752	MG566078_ChiLC	KP235539_ChiLC	Unknown (KP195266__ChiL)	R, G, B, M, C, S, **3S**	5.50 × 10^−29^
17	2174	2516	KU923758__ChiL	HM140366_ChiLC	Unknown (DQ673859_ChiLC)	R, G, M, C, S, **3S**	2.85 × 10^−23^
18	484	920	MT636371_ChiLC	KT699194__ChiL	MH765693__ChiL	R, G, M, C, **S**, 3S	3.80 × 10^−32^
19	2754	481	MT636373_ChiLC	Unknown (JN663852__ChiL)	MK882926_ChiLC	**R**, G, M, C, S, 3S	1.26 × 10^−17^
20	2732	1076	DQ673859_ChiLC	MK882926_ChiLC	Unknown (FM179613__ChiL)	**R**, G, M, C, S, 3S	8.76 × 10^−16^
21	111	513	MF737343_ChiLC	JN604491_ChiLC	MK882926_ChiLC	R, G, M, C, S, **3S**	2.56 × 10^−14^
22	1943	2740	KP195266__ChiL	Unknown (HM007116_ChiLC)	KP235539_ChiLC	R, G, B, M, C, **S**, 3S	4.05 × 10^−21^
23	1069	1302	JN555600__ChiL	Unknown (DQ114477_ChiLC)	MT636371_ChiLC	**R**, G, M, C, S, 3S	9.52 × 10^−14^
24	1751	2036	MH355641_ChiLC	LY564869__ChiL	Unknown (MH765698__ChiL)	**R**, G, B, M, C, S, 3S	1.52 × 10^−12^
25	466	958	FJ345402_ChiLC	KT699194__ChiL	MH765693__ChiL	**R**, M, C, S, 3S	1.78 × 10^−12^
26	509	1393	KU376495_ChiLC	MK882926_ChiLC	Unknown (GU136803_ChiLC)	R, G, B, M, C, **S**, 3S	9.47 × 10^−12^
27	2614	151	KR779820__ChiL	MH765697_ChiLC	Unknown (KX951415__ChiL)	**R**, G, M, C, S, 3S	3.55 × 10^−11^
28	1211	1726	MK882926_ChiLC	HM140371_ChiLC	JN604491_ChiLC	R, G, B, M, C, S, **3S**	8.45 × 10^−12^
29	2747	446	MT636371_ChiLC	KR957353_ChiLC	FJ345402_ChiLC	**R**, G, M, C, S, 3S	6.33 × 10^−8^
30	1807	2205	MH538340__ChiL	MK882926_ChiLC	FM179613__ChiL	R, G, B, M, C, **S**, 3S	4.46 × 10^−8^
31	1091	1540	FM179613__ChiL	HM007104_ChiLC	KT699194__ChiL	**R**, G, M, C, S, 3S	2.94 × 10^−7^
32	1411	2071	DQ114477_ChiLC	KF312364__ChiL	KT699194__ChiL	R, G, B, M, C, **S**, 3S	4.46 × 10^−14^
33	1209	1533	MF737343_ChiLC	KF229719_ChiLC	HM007104_ChiLC	R, G, B, M, C, S, **3S**	8.96 × 10^−9^
34	1761	2131	MH765698__ChiL	MH765697_ChiLC	MK882926_ChiLC	**R**, B, M, C, 3S	8.71 × 10^−07^
35	178	974	MT316405_ChiLC	KF471061_ChiLC	MH765697_ChiLC	R, M, C, **S**, 3S	1.28 × 10^−24^
36	2693	429	MH765693__ChiL	Unknown (JN555600__ChiL)	GU136803_ChiLC	R, G, B, M, C, **S**, 3S	3.85 × 10^−11^
37	76	535	GU136803_ChiLC	KR957353_ChiLC	KT699194__ChiL	**R**, G, B, M, C, S, 3S	5.25 × 10^−6^
38	2693	34	LY564869__ChiL	HM140365_ChiLC	KT699194__ChiL	**R**, G, 3S	1.43 × 10^−5^
39	2088	2205	MT636373_ChiLC	MK882926_ChiLC	KF471061_ChiLC	R, G, B, M, C, **S**, 3S	2.39 × 10^−8^
40	1528	1750	KM880103_ChiLC	MN885878_ChiLC	KF229719_ChiLC	R, G, B, M, C, S, **3S**	1.23 × 10^−6^
41	1128	1527	KM880103_ChiLC	MT636373_ChiLC	KT699194__ChiL	R, G, B, M, C, **S**, 3S	5.94 × 10^−8^
42	1638	2105	KU923757__ChiL	Unknown (HM140370_ChiLC)	HM140364_ChiLC	**R**, G, B, M, C, S, 3S	1.65 × 10^−5^
43	1064	1153	KT699194__ChiL	MT636371_ChiLC	Unknown (FJ345402_ChiLC)	R, **G**, M, S, 3S	1.72 × 10^−5^
44	2086	2203	MK882926_ChiLC	KY420138__ChiL	Unknown (JN604491_ChiLC)	**R**, G, 3S	5.29 × 10^−5^
45	1234	1602	MT636371_ChiLC	HM140370_ChiLC	Unknown (JN604491_ChiLC)	R, B, M, C, S, **3S**	8.58 × 10^−5^
46	68	226	MN417112_ChiLC	JN663846_ChiLC	Unknown (KT699194__ChiL)	R, G, B, **3S**	6.15 × 10^−5^
47	1077	1181	KP195266__ChiL	Unknown (HM007104_ChiLC)	JN604491_ChiLC	R, M, C, **3S**	2.03 × 10^−5^
48	2709	157	KT699194__ChiL	Unknown (KM023147_ChiLC)	HM140370_ChiLC	**R**, G, B, M	4.21 × 10^−4^
49	2651	2738	KP235539_ChiLC	KM023148_ChiLC	HM992939_ChiLC	R, G, **3S**	7.16 × 10^−5^
50	2190	2731	DQ673859_ChiLC	Unknown (KM023148_ChiLC)	HM140366_ChiLC	**R**, S, 3S	1.37 × 10^−3^
51	1853	2200	KR957353_ChiLC	Unknown (HM007114_ChiLC)	KJ700653_ChiLC	R, C, **3S**	4.09 × 10^−3^
**b**							
**Events**	**Breakpoints**		**Recombinant**	**Parents**		**Method ^$^**	***p*-Value ^#^**
	**Begin**	**End**		**Major**	**Minor**		
**Rep protein AC1**							
1	185	511	MH765698	Unknown (MH355641)	AF336806	R, G, M, C, **S**, 3S	8.45 × 10^−35^
2	820	1071	KP868762	Unknown (MN417111)	DQ673859	R, G, M, C, **S**, 3S	1.12 × 10^−13^
3	35	418	DQ114477	Unknown (KP195266)	MN417111	R, G, M, C, S, **3S**	5.34 × 10^−16^
4	218	303	JN555600	MH355641	Unknown (MH475358)	**R**, G, M, C, S, 3S	2.98 × 10^−11^
5	202	562	MH355641	MH538340	Unknown (MK882926)	R, G, M, C, S, **3S**	3.84 × 10^−18^
6	679	77	KY420138	HM140366	Unknown (DQ673859)	R, G, M, C, **S**, 3S	8.61 × 10^−19^
7	631	942	MF737343	MK757217	Unknown (KP195266)	R, G, M, C, S, **3S**	5.10 × 10^−18^
8	1068	741	MG566078	AF336806	MK757217	R, G, M, C, S, **3S**	4.93 × 10^−18^
9	255	573	MK757217	AF336806	Unknown (MK882926)	R, G, M, C, S, **3S**	9.62 × 10^−11^
10	640	1068	AF336806	HM140366	KP195266	R, G, M, C, **S**, 3S	5.82 × 10^−12^
11	735	939	MT636373	MT636371	Unknown (MT316404)	**R**, M, C, S, 3S	1.03 × 10^−4^
12	425	1068	KP195266	Unknown (HM140366)	MN417111	R, M, C, **S**, 3S	9.20 × 10^−11^
13	234	619	KJ700656	HM007114	KU923757	R, G, M, C, S, 3S	4.62 × 10^−7^
14	37	258	MK882926	HM140370	Unknown (KU923758)	R, G, M, **C**, 3S	1.91 × 10^−5^
15	1035	184	MH765698	JN555600	KJ700656	R, **M**, C, 3S	1.30 × 10^−5^
**TrAP protein AC2**							
1	404	88	HM992939	DQ114477	KY420138	R, **G**, M, C, 3S	2.40 × 10^−7^
2	205	315	DQ114477	Unknown (KJ700656)	LN886657	R, G, M, C, **S**, **3S**	1.97 × 10^−8^
3	247	320	KJ700656	DQ116878	KP868762	R, G, M, C, **S**, 3S	3.63 × 10^−8^
**REn protein AC3**							
1	212	406	DQ116878	Unknown (JN555600)	MH538340	R, G, M, C, **S**, 3S	7.81 × 10^−15^
2	106	234	MH765698	MF737343	HM992939	R, M, C, **S**, 3S	1.85 × 10^−13^
3	263	61	MH765693	HM992939	Unknown (JN604491)	R, M, C, **S**, 3S	3.03 × 10^−9^
**C4 protein**							
1	279	175	KP868762	MK882926	NC028046	B, M, **S**, 3S	3.52 × 10^−7^
**CP-protein AV1**							
1	740	228	MF737343	Unknown (MH355641)	KM921669	R, G, M, C, S, **3S**	5.68 × 10^−23^
2	762	314	MT316405	Unknown (MH355641)	KP868762	R, G, M, C, S, **3S**	2.04 × 10^−26^
3	759	225	MH355641	KU923758	Unknown (KP868762)	R, G, M, C, **S**, 3S	4.01 × 10^−11^
4	403	702	KU376495	HM140370	Unknown (HM140366)	R, M, **S**, 3S	1.06 × 10^−8^
5	38	240	MT636373	Unknown (MH538340)	DQ989326	R, **S**, 3S	2.32 × 10^−2^
**Pre-CP protein AV2**							
1	348	128	KM880103	MH355641	Unknown (MG566078)	R, G, M, C, S, **3S**	2.11 × 10^−9^
				**Alphasatellite**			
1	1058	1161	KF471058_ChiLC	KF471037_CHiLC	Unknown (KF471050_ChiLC)	R, G, B, M, C, S, **3S**	5.90 × 10^−11^
2	758	1048	KF471050_ChiLC	Unknown (KF471058_ChiLC)	KF471037_CHiLC	R, B, M, C, **S**, 3S	9.46 × 10^−38^
3	308	624	KF584013_ChiLC	KF471058_ChiLC	Unknown (KF471049_ChiLC)	R, M, C, **S**	1.46 × 10^−14^
4	929	1010	KF471058_ChiLC	KF471037_CHiLC	Unknown (KF584013_ChiLC)	R, **G**, 3S	6.78 × 10^−3^
				**Betasatellite**			
1	1260	1310	MT385290_ChiLC	AM279663_ChiLC	Unknown (JN638446_ChiLC)	**R**, G, B, M, C, 3S	2.30 × 10^−24^
2	141	493	MF737346_ChiLC	KJ614228_ChiLC	MT385300_ChiLC	**R**, G, B, M, C, S, 3S	2.03 × 10^−23^
3	1033	1282	KJ614228_ChiLC	AM279666_ChiLC	MT316408_ChiLC	**R**, G, B, M, C, S, 3S	3.77 × 10^−13^
4	1161	1206	AM279666_ChiLC	LT608340_ChiLC	Unknown (EU582020_ChiLC)	R, **G**, B, M, C, S, 3S	7.57 × 10^−11^
5	1079	1142	AM258978_ChiLC	LT608339_ChiLC	Unknown (KT835647_ChiLC)	R, **G**, M, C, S, 3S	3.73 × 10^−10^
6	723	1242	KU376496_ChiLC	Unknown (KX302717_ChiLC)	MN080501_ChiLC	**R**, G, 3S	1.64 × 10^−9^
7	1342	1377	MN080501_ChiLC	MF737346_ChiLC	Unknown (JN638446_ChiLC)	**R**, G, 3S	8.01 × 10^−8^
8	1293	242	KU376496_ChiLC	Unknown (MH411247_ChiLC)	AM258978_ChiLC	**R**, G, B, M, C, S, 3S	8.15 × 10^−7^
9	243	367	MN080501_ChiLC	MK737916_ChiLC	AJ316032_CHiLC	**R**, G, M, 3S	6.82 × 10^−6^
10	1003	1059	MT800762_ChiLC	Unknown (AM849549_ChiLC)	MK737916_ChiLC	R, G, **S**, 3S	1.22 × 10^−28^
11	975	1073	MK737916_ChiLC	AM279665_ChiLC	Unknown (AJ316032_CHiLC)	**R**, G, M, C	2.33 × 10^−5^
12	1077	1248	MT385293_ChiLC	FM877803_ChiLC	KT835647_ChiLC	R, **G**, B, M, 3S	3.97 × 10^−7^
13	1019	1100	MT385290_ChiLC	AM279666_ChiLC	Unknown (EU582020_ChiLC)	R, G, **S**, 3S	4.00 × 10^−26^
14	1087	1338	KP892648_ChiLC	DQ343289_ChiLC	Unknown (MT385294_ChiLC)	R, M, C, **3S**	2.93 × 10^−5^
15	323	453	MT385293_ChiLC	FM179615_ChiLC	Unknown (MT385300_ChiLC)	**R**, B, M, 3S	5.34 × 10^−4^
16	960	1138	KX302717_ChiLC	FM877803_ChiLC	MT385300_ChiLC	R, **M**, 3S	4.45 × 10^−5^
17	148	232	MT385294_ChiLC	MT385295_ChiLC	LT827053_ChiLC	R, **S**, 3S	6.43 × 10^−4^

^$^ R-RDP; G-Geneconv; B-Bootscan; M-MaxChi; C-Chimarea; S-SiScan; 3Seq-Sequence Triplets; ^#^ The lowest *p*-value calculated for the underline and bold method are given in the column.

**Table 3:** Genetic diversity of ChiLCV DNA-A, along with all six genes and satellite molecules.

Virus Component	η	π	k	θ - η	θ - W	H	Hd
**DNA-A**	2104	0.10769	257.81691	0.16108	0.10817	113	0.997
**CP**	563	0.12144	90.11150	0.13906	0.09806	81	0.977
**Pre-CP**	305	0.13907	47.14315	0.22340	0.22340	55	0.998
**Rep**	399	0.17521	48.18240	0.26592	0.16195	68	0.927
**TrAP**	426	0.14246	35.18726	0.31744	0.17660	72	0.965
**REn**	282	0.15639	32.37376	0.25038	0.15627	70	0.972
**C4**	322	0.18375	39.32350	0.27577	0.16187	62	0.922
**Alphasatellite**	727	0.13886	178.29524	0.17413	0.13772	14	0.990
**Betasatellite**	1070	0.10878	109.43568	0.21760	0.14317	66	0.996

**Table 4:** Results of different neutrality tests and selection pressure analysis for DNA-A with each ORFs and satellites.

Virus Component	dN	dS	dN/dS	Total Number of Amino Acid Sites under Positive Selection	Neutrality Tests		
					Tajima’s D	Fu and Li’s D	Fu and Li’s F
**DNA-A**	0.066 ± 0.003	0.064 ± 0.003	1.03125	**101**	−1.105	−1.970	−1.869
**CP**	0.073 ± 0.006	0.065 ± 0.006	1.12307	**2**	−0.420	−1.192	−0.997
**Pre-CP**	0.043 ± 0.008	0.038 ± 0.011	1.13157	**1**	−1.453	−1.918	−2.086
**Rep**	0.134 ± 0.008	0.299 ± 0.015	0.44816	**17**	−1.127	−0.128	−0.706
**TrAP**	0.094 ± 0.009	0.133 ± 0.018	0.70676	**13**	−1.826	−1.794	−2.165
**REn**	0.128 ± 0.019	0.167 ± 0.023	0.76646	**5**	−1.237	0.012	−0.670
**C4**	0.127 ± 0.015	0.200 ± 0.028	0.635	**6**	−1.100	1.158	0.141
**Alphasatellite**	0.093 ± 0.006	0.086 ± 0.005	1.08139	**3**	−0.902	−0.964	−1.092
**Betasatellite**	0.072 ± 0.004	0.055 ± 0.003	1.30909	**34**	−1.750	−2.557	−2.664

## Data Availability

All sequencing data of virus isolates are available in the NCBI database. Further data analysis will be available from the corresponding authors upon request.

## Supplementary Materials

The following supporting information can be downloaded at: https://www.mdpi.com/article/10.3390/pathogens11050529/s1, Supplementary Figures S1–S3: Pairwise identity matrix of all ChiLCV DNA-A (S1) and associated satellites sequences for alpha (S2) and beta (S3) inferred using Species Demarcation Tool (SDT) v1.2.
