# Supplementary figures and images for: Role of Diversity and Recombination in the Emergence of Chilli Leaf Curl Virus
Source: Pathogens. 2022 Apr 30;11(5):529. doi: 10.3390/pathogens11050529 (PMC9146097; doi:10.3390/pathogens11050529)

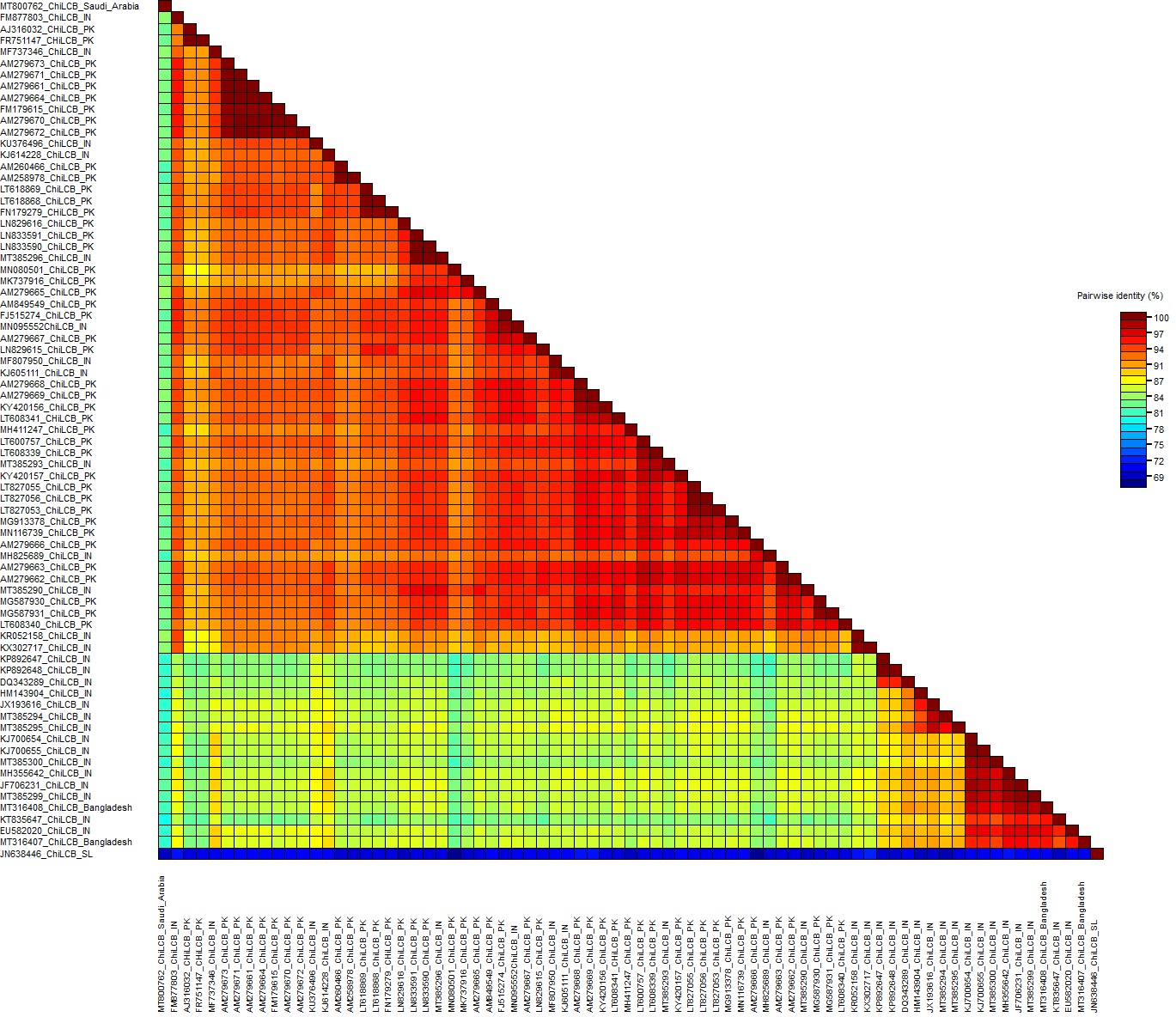

Supplement: Supplementary file 1 [file pathogens-11-00529-s001.zip › Supplementary Figure S2.jpg]

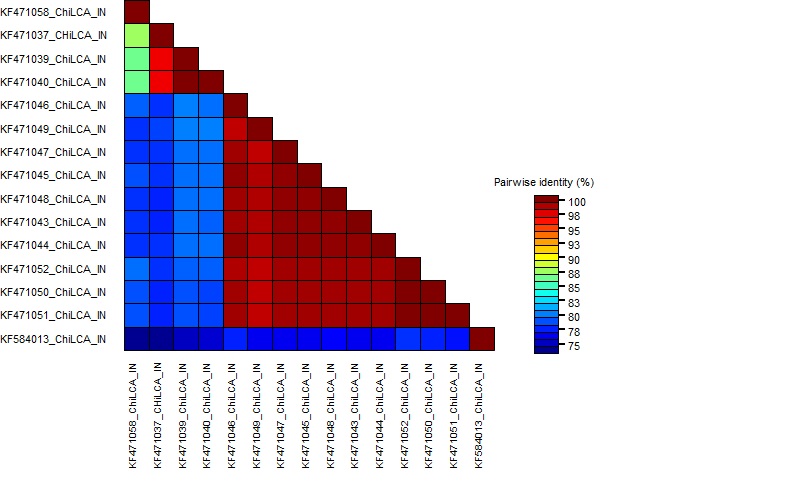

Supplement: Supplementary file 1 [file pathogens-11-00529-s001.zip › Supplementary Figure S3.jpg]

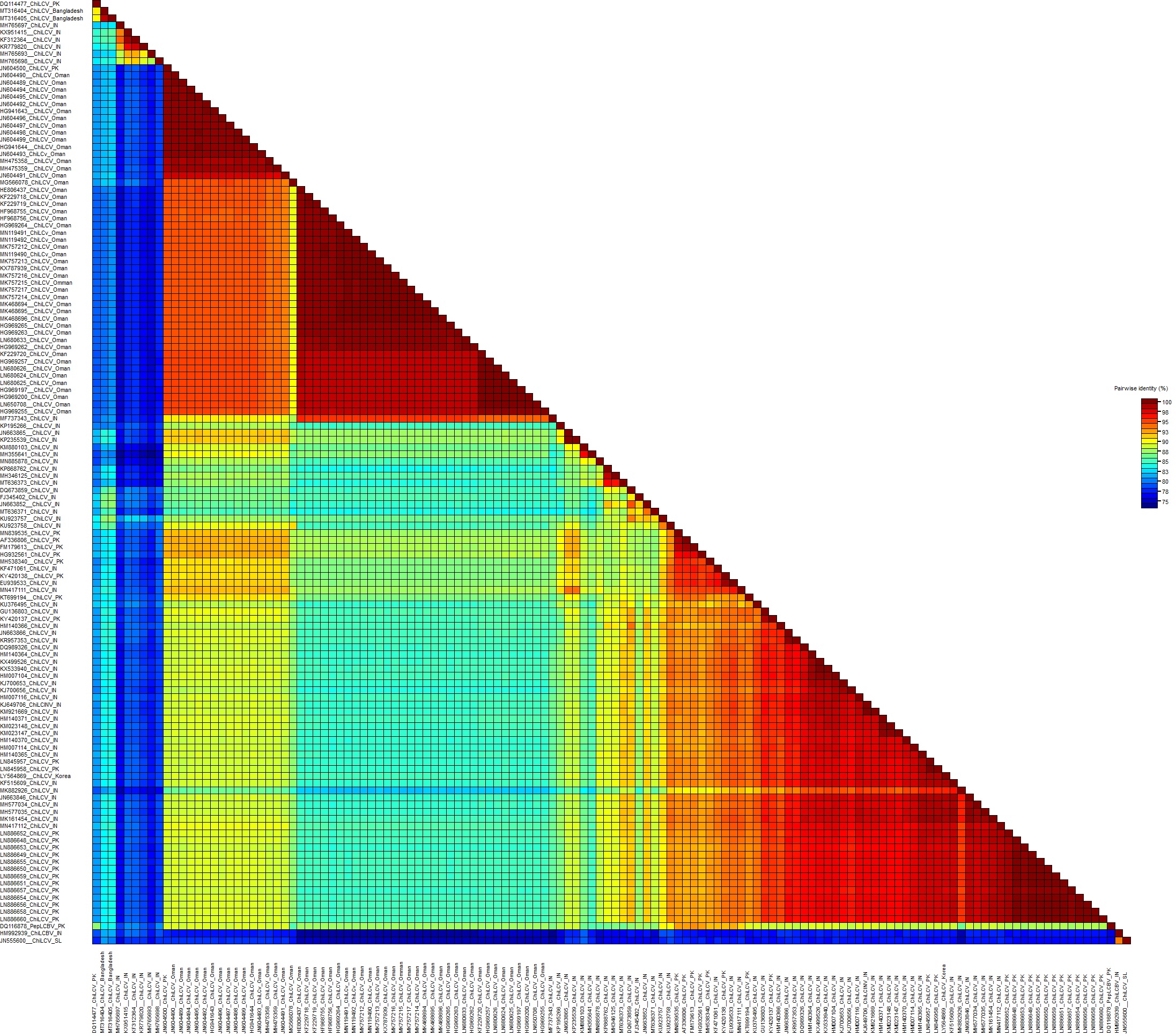

Supplement: Supplementary file 1 [file pathogens-11-00529-s001.zip › Supplementary.Figure.S1.jpg]
